# Patient safety in community-based teaching hospitals

**DOI:** 10.3402/jchimp.v1i2.6433

**Published:** 2011-07-18

**Authors:** Ethan D. Fried

**Affiliations:** Department of Internal Medicine, St. Luke's-Roosevelt Hospital, New York, NY, United States

It has been over 10 years since the Institute of Medicine (IOM) re-ignited the patient safety movement in the United States with their publication ‘To Err is Human’. Five years after that publication there were modest improvements in certain indicators like death in low mortality Diagnosis Related Groups (DRGs), iatrogenic pneumothorax, and certain postoperative complications. At that time there was actually worsening in the rates of hospital acquired decubitus ulcers and postoperative deep vein thromboses and pulmonary emboli (1). Five years after the IOM report, leaders in patient safety such as Lucien Leape, Donald Berwick (2), and Robert Wachter (3) described safety systems that were then in their infancy (or at best early adolescence). These included specific tools like electronic health records and computerized physician order entry systems, as well as safe practices like improved team training, sharing of best practices, and full disclosure to patients following injuries. Strategies to encourage the development of these systems included ‘pay for performance’ schemes, non-payment for ‘never events’, regulation and accreditation, better reporting systems, funding for health information technology, malpractice reform, and workforce and training systems were identified. In January 2010, Robert Wachter added to this list of safety reform systems additional elements (4). Some of which were identified in the IOM report. These were policies to promote safety research, better ways to engage patients and their families in the avoidance of errors, internal leadership, and the creation of business models that encouraged safety and national and organizational support (5).

While all investigators have reported slow but steady gains in patient safety over the past decade, a disturbing trend has persisted. The 2009 Healthgrades report makes it clear that the chasm between the performance of the country's best hospitals and that in all other hospitals is widening. This is one example of how the rising tide has not raised all boats equally. In order to achieve industry-wide improvement and to re-characterize our profession as one that effectively learns from our mistakes, we must democratize our safety systems and incorporate them into the culture of all of our organizations and not just the elite few. In this issue of the *Journal of Community Hospital Internal Medicine Perspectives* there are reports on specific systems to improve patient safety. The systems cover hypoglycemia, antibiotic stewardship, and hand hygeine. These systems are being implemented in community-based hospitals just as they are in university-associated teaching hospitals. They have the potential to save lives by transforming even average hospitals into what Amalberti et al. have termed ‘highly reliable organizations’ (6). Highly reliable organizations are adaptive and use practice guidelines when available but have experts on hand to challenge the boundaries of their own performance. These organizations work to standardize practice and improve the links between departments and between the various roles involved in patients’ care. Excellent communication and disciplined use of evidence-based systems to improve patient safety can be achieved by any organization regardless of size and governance structure.

In New York State, the Department of Health and the New York Chapter of the American College of Physicians have been monitoring near miss medical errors for the past several years. The ‘NYS Near Miss Registry’ has observed some of the same phenomena as have been pointed out in Healthgrades and other reports. This is the observation that institutions that have incorporated various safety systems into their culture are seeing fewer specific types of errors. In other words, evidence-based barriers really work to reduce specific types of errors. Our database allows us to collect information on near misses on a risk free and anonymous web-based survey. Without identifying the institution involved, we can collect demographic information on safety systems that are in place at that institution. Verbatim descriptions of the error and the barrier that prevented it from reaching the patient can then be coded into a standard set of keywords that help us to characterize both the safety vulnerabilities in that institution as well as the effectiveness of medical error barriers and precautions. What we have discovered is that while risks seem to persist at all institutions regardless of the sophistication of its safety systems, the safety barriers work well where they are in place. Institutions without sophisticated barriers such as computerized physician order entry, medication bar coding, electronic health records, reliable standardized hand-off systems, and work-flow systems that limit fatigue in residents rely entirely on individuals (residents, nurses, pharmacists, etc.) to identify patient risks and avoid errors. It is not hard to see that systems are more reliable than individuals in maintaining vigilance and prevent errors from reaching patients.

The achievement of safe medical care and the creation of a highly reliable organization do not have to depend on the size or governance of an institution. Relatively small projects like the incorporation of a ventilator bundle can reduce the incidence of ventilator-associated pneumonia and reduce the overall utilization of intensive care resources in a community hospital environment (7). A comprehensive medication safety system can similarly reduce adverse events (8). Systems that promote simple precautions like hand washing and strict isolation of *c.difficile* infected patients save hospital days and save lives in any environment. Strong medication reconciliation practices clearly reduce adverse events, prevent re-admissions, and make post-hospital care more effective. Disciplined standardized hand off systems streamline care and avoid adverse events related to pain control, drug interactions, use of medications to which the patient is allergic, anticoagulation medication related errors, and others. These systems do not require a medical school to implement. Any community-based hospital with a commitment to patient care that is safe, effective, efficient, timely, patient-centered, and equitable can implement these steps.
